# Treatise on Inflammation as a Process of Anormal Nutrition

**Published:** 1845-01

**Authors:** 


Art. VI.—*Treatise on Inflammation as a process of Anormal Nutrition.* By J. H. Bennett, m.d. f.r.s.e. &c. &c.—*Edinb.* 1844. 8vo, pp. 80.

This treatise, we are informed, is the substance of six lectures delivered at the evening meetings of the Royal College of Physicians in Edinburgh, in the summer of 1843.

Dr. Bennett discusses his subject under the following heads: 1st, cellular theory of nutrition; 2d, the blood; 3d, the capillaries; 4th, the early phenomena of inflammation; 5th, the essential phenomena of inflammation; 6th, terminations of inflammation; 7th, the circumstances influencing the terminations; and lastly, the conclusions from the whole.

Dr. Bennett's leading view is that an anormal exudation of blood-plasma constitutes the essential phenomena of inflammation. In this he accords with the views which have been recently expressed by ourselves; and we believe that the tendency to found upon it the essential distinction between congestion and inflammation is gaining ground among intelligent pathologists.

The subject of inflammation has of late been so fully discussed in this Review, that it is unnecessary to dwell on it on the present occasion. There are a few points, however, in the treatise before us, which Dr. Bennett calls attention to, as not having been noticed before. They are the identity in structure between the capillaries and non-voluntary muscular fibre; the structure of lymph in its various forms; the distinction between the plastic and exudative corpuscles; the identity of the latter with the granular bodies of the colostrum; and the means of distinguishing inflammatory and non-inflammatory softening.

In regard to the identity in structure between the capillaries and non-voluntary muscular fibre, we must confess that we have not been able to follow Dr. Bennett's demonstration of it. The contractility of the wall of the true capillary has of late been an open question, and we think that it still remains so from anything that Dr. Bennett has adduced on the point. Here we may remark that, in speaking of the resemblance between the fibres of the middle coat of arteries and of the muscular coat of the intestines, Dr. Bennett overlooks what Henle has done and said on the subject, and as to the contractility of arteries we do not think that Dr. Bennett correctly represents the present opinions of physiologists and pathologists on the point, when he attributes to them the opinion that the arteries are not contractile.

As to the other points referred to, we shall not stop to inquire into their novelty; it is enough to remark that Dr. Bennett discusses them very well. His lectures may be advantageously consulted by those who desire to become acquainted with the various recent contributions—foreign as well as British—to our knowledge of the subject.

